# Editorial: Antimicrobial resistance in dairy and poultry production: challenges and solutions

**DOI:** 10.3389/fvets.2026.1792063

**Published:** 2026-02-09

**Authors:** Vinod Kumar Singh, Vivek Kumar Singh, Amit Kumar Singh, Chayanika Das

**Affiliations:** 1 Department of Veterinary Microbiology, College of Veterinary and Animal Sciences, Rani Lakshmi Bai Central Agricultural University (RLBCAU), Jhansi, UP, India; 2 Department of Veterinary Physiology and Biochemistry, College of Veterinary and Animal Sciences, Rani Lakshmi Bai Central Agricultural University (RLBCAU), Jhansi, UP, India; 3 Albany Medical College, Albany, NY, United States; 4 Institute of Para Veterinary Sciences, Uttar Pradesh Pandit Deen Dayal Upadhyaya Pashu Chikitsa Vigyan Vishwavidyalaya Evam Go Anusandhan Sansthan (DUVASU), Mathura, UP, India

**Keywords:** AMR, dairy, pharmacology, poultry, veterinary science

The surging population and declining amount of arable land coupled with rising consumer demand for affordable and high-quality food have driven the emergence of intensive dairy and poultry production systems. Antibiotics are being used extensively in livestock and poultry industries for treatment, disease prevention, and growth promotion to cater to the ever-growing demand (1, 2). Unfortunately, the excessive and uncontrolled use of antimicrobials has led to the development of antimicrobial resistance and wide dissemination of resistant bacteria, resistance genes, and residues, thereby jeopardizing ecosystem health (3, 4). Currently, antimicrobial resistance (AMR) is a critical threat to public health, sustainable food systems, and livelihoods globally (5). Therefore, recognizing the role of dairy and poultry production systems in the critical nexus of emerging AMR debate, this Research Topic was conceived to propose mitigating strategies to safeguard antimicrobial efficacy while ensuring sustainable production from learned minds across the globe.

This Research Topic provides a comprehensive collection of articles that focus on the evidence-based drivers of AMR and realistic mitigating strategies in dairy and poultry systems. The contributions emphasize that AMR is a complex One-Health problem often driven by microbiological, pharmacological, socio-economical, environmental, technological, and regulatory factors. Collectively, the articles illustrate how mitigating strategies depend on integrating antimicrobial stewardship, surveillance, alternatives to antibiotics, behavioral and policy research, and advances in veterinary pharmacology. This Research Topic also highlights the persistent gap between awareness of AMR and the implementation of sustainable interventions, particularly in low-to-middle-income economies. In a systematic review, Jacobsen et al. underscores the lack of well-researched and evidence-based AMR interventions in the livestock systems of sub-Saharan Africa. The reported interventions were mainly focused on enhancing awareness, knowledge, or attitudes rather than any measurable real-world outcome. Importantly, this review emphasizes that adoption of any intervention heavily depends on economic constraints, perceived sustainability, and organizational culture. In an experimental study, Al-Khalaifah et al. have provided an evidence-based, credible, and scalable alternative to antimicrobials in poultry systems. The combination of bacteriophages with phytochemicals not only controlled multidrug-resistant *Salmonella* Typhimurium but also improved growth performance, gut health, and immune responses. Such integrated approaches can enhance productivity while keeping the selection pressure for resistance in check. This shows that productivity and AMR control are not mutually exclusive: a well-designed strategy can achieve both in poultry as well as in dairy systems. Another experimental study on Ugandan poultry by Wainaina et al. revealed the detection of plasmid-mediated colistin resistance (*mcr*-1), identifying clonally related, multidrug-resistant *Escherichia coli* carrying transmissible resistance elements. Despite a low prevalence, the study underscores the risk of AMR to critically important antimicrobials. This study reinforces the urgency of robust surveillance of antimicrobial use and resistance in poultry and dairy systems to formulate any evidence-informed policy against AMR. While studying other factors contributing to farm-level AMR, Adegbole et al. list constraints faced by researchers. The factors are inadequate access to farms, trust deficits, inconsistent metadata, and limited data-sharing frameworks. Challenges due to inaccurate or inaccessible data draw attention to the need for strengthened data governance, transparent partnerships, and standardized protocols to develop actionable stewardship and prudent strategies. Innovation in pharmacology is crucial in adopting rational use of antimicrobials. In this Research Topic, Sun et al. demonstrated that the use of enrofloxacin encapsulated in polymeric micelle significantly improved solubility, bioavailability, and efficacy. Such innovations help in reducing treatment dose, duration, and the withdrawal period, thus minimizing resistance selection and residue risk. Such advances are especially relevant to both dairy and poultry medicine, where optimized formulations may allow lower effective doses, reduced treatment duration, and improved compliance with withdrawal periods—factors that collectively contribute to minimizing resistance selection and residue risks.

As editors of this Research Topic, we wish to convey a central message that the mitigating strategies of AMR in dairy and poultry production systems demand an orchestrated action plan across multiple disciplines, sectors, and levels of governance. Robust surveillance, transparent data management, and adoption of innovation, while taking into account the socio-economic and environmental factors of the region, may help in designing prudent strategies to combat AMR in poultry and dairy production systems. This can be achieved with the active and synchronized involvement of stakeholders like producers, researchers, funders, and policymakers. Importantly, capacity building, awareness programs, partnerships, and technology transfer matched with enabling policies must be met with effective stewardship frameworks and sustained investment. Looking ahead, we believe that progress should be monitored and tailored to achieve results in diverse dairy and poultry production systems. Conclusively, we believe that the AMR mitigating strategies can be addressed if future research is focused on intervention studies that demonstrate efficient reduction in resistance while accounting for economic feasibility at the farm level. We hope readers will find this Research Topic informative and that the Research Topic will stimulate interdisciplinary collaboration in the formulation of evidence-informed policies to protect animal, public, and environment health as well as the sustainability of global dairy and poultry production systems.
